# A Pilot Study of Musculoskeletal Abnormalities in Patients in Recovery from a Unilateral Rupture-Repaired Achilles Tendon

**DOI:** 10.3390/ijerph17134642

**Published:** 2020-06-28

**Authors:** Dong Sun, Gusztáv Fekete, Julien S. Baker, Qichang Mei, Bíró István, Yan Zhang, Yaodong Gu

**Affiliations:** 1 Faculty of Sports Science, Ningbo University, Ningbo 315211, China; nbsundong@gmail.com (D.S.); meiqichang@aliyun.com (Q.M.); champagne0906@hotmail.com (Y.Z.); 2 Savaria Institute of Technology, Eötvös Loránd University, 9700 Szombathely, Hungary; fg@inf.elte.hu; 3 Department of Sport and Physical Education, Hong Kong Baptist University, Hong Kong 999077, China; jsbaker@hkbu.edu.hk; 4 Department of Technology, Faculty of Engineering, University of Szeged, 6727 Szeged, Hungary; biro-i@mk.u-szeged.hu

**Keywords:** Achilles tendon rupture, musculoskeletal modeling, knee, ankle, gait

## Abstract

The purpose of this study was to compare the inter-limb joint kinematics, joint moments, muscle forces, and joint reaction forces in patients after an Achilles tendon rupture (ATR) via subject-specific musculoskeletal modeling. Six patients recovering from a surgically repaired unilateral ATR were included in this study. The bilateral Achilles tendon (AT) lengths were evaluated using ultrasound imaging. The three-dimensional marker trajectories, ground reaction forces, and surface electromyography (sEMG) were collected on both sides during self-selected speed during walking, jogging and running. Subject-specific musculoskeletal models were developed to compute joint kinematics, joint moments, muscle forces and joint reaction forces. AT lengths were significantly longer in the involved side. The side-to-side triceps surae muscle strength deficits were combined with decreased plantarflexion angles and moments in the injured leg during walking, jogging and running. However, the increased knee extensor femur muscle forces were associated with greater knee extension degrees and moments in the involved limb during all tasks. Greater knee joint moments and joint reaction forces versus decreased ankle joint moments and joint reaction forces in the involved side indicate elevated knee joint loads compared with reduced ankle joint loads that are present during normal activities after an ATR. In the frontal plane, increased subtalar eversion angles and eversion moments in the involved side were demonstrated only during jogging and running, which were regarded as an indicator for greater medial knee joint loading. It seems after an ATR, the elongated AT accompanied by decreased plantarflexion degrees and calf muscle strength deficits indicates ankle joint function impairment in the injured leg. In addition, increased knee extensor muscle strength and knee joint loads may be a possible compensatory mechanism for decreased ankle function. These data suggest patients after an ATR may suffer from increased knee overuse injury risk.

## 1. Introduction

Achilles tendon ruptures (ATR) are common injuries among adults and have compounding long-term effects. Over the past 30 years, the incidence of ATR has been increasing from 2 to 22 per 100,000 people per year, primarily in the athletic population [1]. ATR is more common in males than in females with the ratio being quoted at 5:1 [2]. Tendons are not contractile tissues, and as such cannot provide the necessary positive work to drive human motion. Evidence has been reported that ATR will cause persistent tendon elongation and is associated with biomechanical deficits such as decreased plantar flexor muscle strength/volume, decreased ankle joint proprioception and reduced AT stiffness [3,4].

These functional deficits are reported regardless of the medical efforts and treatment modality for both short-term and long-term outcomes [5,6]. The elongated tendon combined with decreased muscle force and tendon stiffness has been shown to have a substantially decreased running and jumping performance, and also has implications for daily functional activities such as normal gait and walking on stairs [7]. The alterations in calf muscle anatomical, mechanical and neuromuscular properties after ATR may increase plantar flexor muscle activity during movement, leading to AT weakness in a plantarflexed position and reduce plantar flexor endurance [8].

The rupture injury mechanism of ATR can be attributed to sudden unexpected powerful dorsiflexion of the ankle joint or forced dorsiflexion of the plantarflexed ankle joint [9]. A case study reported that long-term functional deficits after ATR could be explained by permanent reductions in gastrocnemius muscle fascicle length [10]. The muscle-tendon structure is detrimentally affected following ATR and the calf muscle strength deficits are positively correlated with the fascicle length reduction magnitude [11]. The atrophy and eccentric strength impairment of calf muscles were still present after 24 months of ATR, which brings adaptive modifications in gait strategy involving ankle motion and coordinated muscular activities [12]. The landing pattern (rearfoot strike vs. non-rearfoot strike) also has an impact on the Achilles tendon loading and a previous study found that people with a non-rearfoot strike running pattern experienced nearly 11% greater Achilles tendon impulse during each step [13]. To further improve treatment, rehabilitation, and enhancing the understanding of the long-term outcome, it is important to understand the underlying mechanisms for musculoskeletal deficits.

Biomechanical analysis is always carried out using experimental or computational methods. The traditional biomechanical analysis based on laboratory experiments has two inadequacies: (1) a lot of important information, including muscle forces, is difficult to measure in the laboratory; (2) separate laboratory data proves difficult to build a relationship with complex human dynamic systems. OpenSim is an open-source software package developed for musculoskeletal model building simulation, and analysis without surgically-invasive procedures [14]. In silico OpenSim models provide a modeling environment for identifying individual variables (kinematics, kinetics, muscle-tendon parameters) and predict muscle force contributions and joint contact loads during walking and running [15].

Extensive research has developed “generic” lower limb musculoskeletal models using cadaveric data. Medical images (ultrasound, computed tomography, magnetic resonance imaging, etc.,) may help generate a high level of anatomical personalization for creating image-based subject-specific musculoskeletal models [16]. Several studies using image-based subject-specific models have been created to estimate knee/tibiofemoral articular forces. The comparison of generic and subject-specific models have also been shown to be more accurate in the assessment of knee joint contact force using subject-specific anatomical parameters [17,18]. To date, few studies have investigated the rupture-repaired Achilles tendon (AT) subject-specific model for estimating muscle forces and joint articular forces. The identification of muscle-tendon parameters, i.e., tendon slack length lst and optimal fiber length lom should be critical aspects when creating subject-specific musculoskeletal models. This is important due to the sensitivity of muscle estimated forces to the muscle-tendon complex moment arms [19].

Based on our prior study, gait asymmetry exists among participants of unilateral Achilles tendon rupture after surgical repair [20]. However, only limited information about how the muscular contribution to walking and running tasks were reported. This study aims to: (a) Build dynamic subject-specific rupture-repaired AT lower limb musculoskeletal models based on ultrasound medical imaging and motion capture data; (b) Compare lower extremity joint angles, moments, quadriceps femoris and triceps surae muscle forces and joint reaction forces between inter-limb (involved vs. uninvolved side) of unilateral rupture-repaired AT patients at two years after surgery during normal walking, jogging and running. It was hypothesized that the gastrocnemii and soleus AT lengths of the involved side will be significantly longer than the uninvolved side. It was further anticipated that the additional slack of the tendon will reduce force transmission across the joint, thus the muscle forces will compensate any increases while the joint reaction forces will decrease.

## 2. Materials and Methods

### 2.1. Subject Recruitment

Six male adults with unilateral rupture surgically repaired AT were included in this two-year follow-up study (26.75 ± 3.71 years, 1.83 ± 0.06 m, 84.2 ± 7.68 kg). Five of the subjects had right side ruptures and one of the subjects had a left side rupture, all the subjects were right-leg dominant. The inclusion criteria for the ATR subjects were between 20–40 years of age with no re-rupture or AT injury on the contralateral side, all the feet types of the participants are normal, without club foot and pes planus foot. The exclusion criteria were physical or neurological conditions and problems existing preventing the subjects from walking normally. The Achilles tendon total rupture score (ATRS) was used for measuring the patients’ symptoms and physical activity levels [21]. The range of ATRS was from 0 to 100, with lower scores indicating greater non-compliance with physical activity and more muscular impairments. The average (SD) ATRS for the six subjects was 87.3 (6) two-years after surgery. All six patients had closed mid-substance ruptures, and this diagnosis was based on the medical examination, including a positive Thompson test and a palpable gap. The patients were given surgical treatment with a modified Kessler technique immediately after the ATR [22]. All the subjects were informed about the purpose of the study and experimental procedures and gave written informed consent. Ethical approval was obtained from the Research Academy of Grand Health, Ningbo University in Ningbo, China on 2018/09/29 (No. 2018RAGH1018).

### 2.2. Achilles Tendon Length Measurement

AT length was taken with a 6.0 cm long linear transducer, scanning frequency of 10 MHz, distance resolution of 0.26 mm ultrasound imaging system (Aloka, Tokyo, Japan). In this study, gastrocnemii and soleus AT lengths were measured from the ultrasound images based on procedures of previous studies [23]. The length of gastrocnemii AT was defined from the calcaneal tubercle (AT insertion point) to the AT junction of medial and lateral gastrocnemii muscles. The length of the soleus AT length was defined from the calcaneal tubercle to the distal end of soleus muscle as previously described in the literature. The subjects were asked to lay in a prone position with their legs fully extended, and the ankle joints were in a neutral position [24]. The ImageJ ultrasound imaging analysis software (version 1.48, Wayne Rasband, Bethesda, MD, USA) was used for measuring the gastrocnemii and soleus AT lengths on both legs.

### 2.3. Experimental Protocol and Procedure Maryland

**Figure 1:**
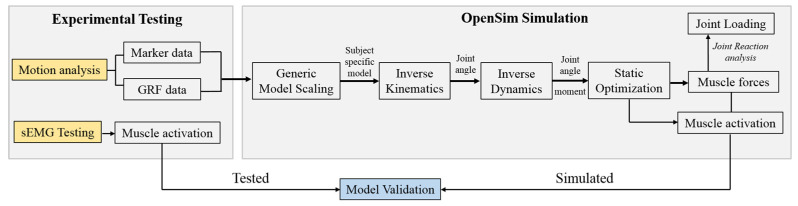
Data processing flow chart, from experimental testing to OpenSim simulation. Note. GRF: ground reaction force; sEMG: surface electromyography.

After warmup and lab familiarization, subjects were outfitted with a validated 37 retro-reflective spherical marker set placed on anatomic bony landmarks with kinematic data collected with an 8-camera motion capture system at 200Hz (VICON Metrics, Oxford, UK). Ground reaction force measurement was synchronized by an in-ground force platform at 1000 Hz (AMTI, Watertown, MA, USA) [25]. Subjects were asked to walk, jog and run barefoot at self-selected speed across a 20m long runway. The subjects were asked to land on the force plate and eight successive trials for every single right and single left foot strike conditions were collected bilaterally on all subjects. The marker trajectory was low-pass filtered using a frequency of 6Hz and the ground reaction forces were filtered at 12Hz using a zero-phase fourth-order Butterworth filter. Marker trajectory data file (.trc) and ground reaction force data file (.mot) were outputted using VICON Nexus software (version 2.8.5, Vicon Motion Systems Ltd., Oxford, UK). Data processing was performed in OpenSim (version 3.3, Stanford University, Stanford, CA, USA) as shown in the workflow (Figure 1). Surface electromyography (sEMG) was recorded for one representative male subject during the stance phase of walking, jogging and running (Delsys, Boston, MA, USA). Measurements were taken from the rectus femoris, biceps femoris long-head (lh), medial gastrocnemius and lateral gastrocnemius at a frequency of 1000 Hz, band-pass filtered between 16 to 380 Hz, and was full-wave rectified. A low-pass filter at a frequency of 10Hz was used to smooth the data. The maximal voluntary isometric contraction (MVIC) trials were performed followed by the trials as reference data. EMG intensities for each muscle were normalized to the MVIC EMG activity from zero to one [26].

### 2.4. Musculoskeletal Modeling

**Figure 2:**
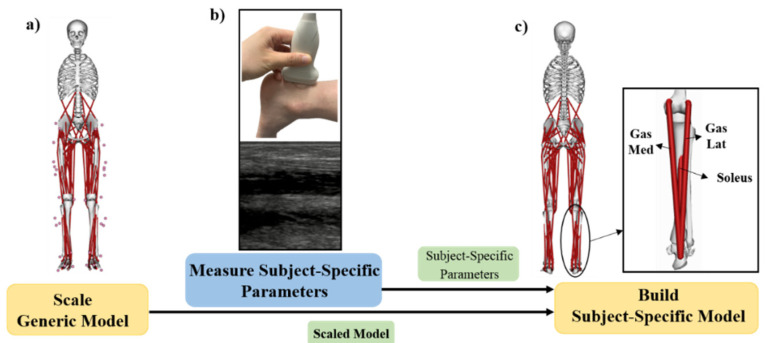
Schematic depiction of the subject-specific musculoskeletal modeling used in this study. (**a**) The generic musculoskeletal model is scaled for each subject using experimental markers placed on anatomical landmarks. (**b**) The slack Achilles tendon length of the involved side was measured with an ultrasound of each subject to obtain subject-specific parameters. (**c**) Graphic depiction of modifying gas_med, gas_lat and soleus muscle-tendon parameters. Note. Gas Med: gastrocnemius medialis, Gas Lat: gastrocnemius lateralis.

An OpenSim Gait 2392 generic musculoskeletal model with 23 degrees of freedom of the lower limbs, pelvis, torso, and head and 92 muscle-tendon actuators without any ligaments was scaled for each participant based on anthropometric characteristics. Muscle attachment points, segment inertial properties, joint articulations, and muscle lengths were scaled to match each participant’s anthropometric profile. Then each participant’s scaled model was modified to create a subject-specific model by specifying the lower limb muscle-tendon architectural parameters based on the subject-specific AT length (ATL) between the involved side (IS) and uninvolved side (US), Figure 2. The muscle fiber length was not measured in this study, while a previous study has shown that if the ATL increases, the muscle length must decrease by the same amount [8]. The optimal fiber length *l_o_^m^* and tendon slack length *l_s_^t^* of the three AT related muscles, including gastrocnemius medialis (MG), gastrocnemius lateralis (LG) and soleus (SL) were proportionally adjusted based on the AT length ratio between the IS and the US, using the equations shown below:

(1) $$\frac{IS\;l_{o}^{m}}{US\;l_{o}^{m}}=\frac{US_{ATL}}{IS_{ATL}}$$

(2) $$\frac{IS\;l_{s}^{t}}{\;US\;l_{s}^{t}}=\frac{IS_{ATL}}{US_{ATL}}$$

In Equations (1) and (2), IS *l_o_^m^* is the optimal fiber length in the involved side, and US *l_o_^m^* is the optimal fiber length in the uninvolved side. IS *l_s_^t^* is the tendon slack length in the involved side, and US *l_s_^t^* is the tendon slack length in the uninvolved side, shown in Table 1. Muscle-tendon parameters modified using the two equations resulted in consistently longer *l_s_^t^* and shorter lom on the IS compared to the US. The joint kinematics were computed using Inverse kinematics (IK) tool, which tracks skin markers and minimizes errors between experimental maker trajectory and the virtual marker set at locations corresponding to the experimental marker in the model. The joint moments were computed with the inverse dynamics (ID) OpenSim tools. The body and joint reference systems were defined using bone geometries based on the ISB conventions. A residual reduction algorithm (RRA) was used to minimize inconsistencies from modeling consumption and experimental testing. A static optimization (SO) approach was used to estimate the muscle force and activation. The SO method minimizes the sum of squared muscle activation at each time step. The muscle weighing constants were modified as 2 for the hamstrings, 1.5 for the gastrocnemius and 1 for the other muscles in this study to reduce muscle forces prediction error based on previous protocols [27,28]. The OpenSim’s JointReaction analyses algorithm was used to calculate the hip, knee and ankle joint forces in this study, which compute the internal joint forces using muscle forces and external and internal loads applied to the model [29].

### 2.5. Model Validation and Sensitivity

To evaluate the model reliability and sensitivity, the recording sEMG was qualitatively compared with the model-simulated muscle activations. The calculated root mean square (RMS) of the four muscles during the walking, jogging and running trails were normalized to the maximum RMS from zero to one during the MVIC testing. 

**Figure 3:**
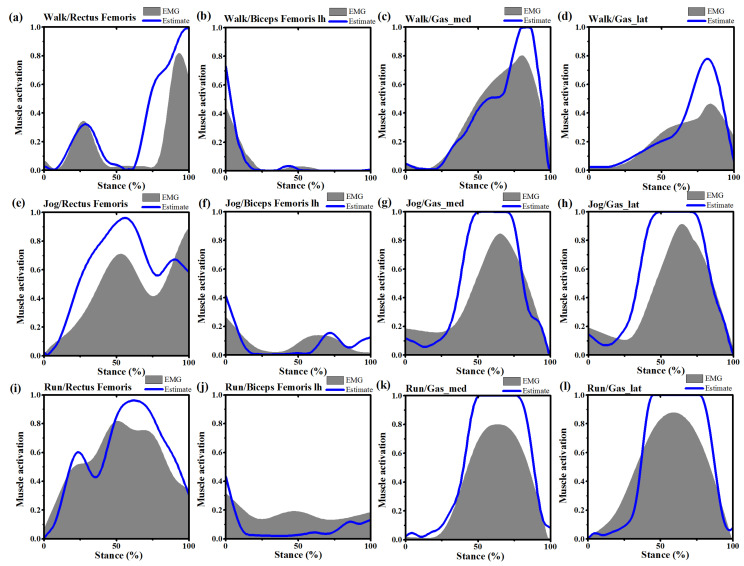
Comparison of muscle activations from static optimization estimated (blue line) and filtered electromyography (EMG) signals measured from one subject during the same trial of normal walking, jogging and running. (**a**): Rectus femoris muscle activation during stance phase of walking; (**b**): Biceps femoris lh (long head) muscle activation during stance phase of walking; (**c**): Gastrocnemius medialis (Gas_med) muscle activation during stance phase of walking; (**d**): Gastrocnemius lateralis (Gas_lat) muscle activation during stance phase of walking; (**e**) Rectus femoris muscle activation during stance phase of jogging; (**f**) Biceps femoris lh (long head) muscle activation during stance phase of jogging; (**g**) Gastrocnemius medialis (Gas_med) muscle activation during stance phase of jogging; (**h**): Gastrocnemius lateralis (Gas_lat) muscle activation during stance phase of jogging; (**i**): Rectus femoris muscle activation during stance phase of running; (**j**): Biceps femoris lh (long head) muscle activation during stance phase of running; (**k**): Gastrocnemius medialis (Gas_med) muscle activation during stance phase of running; (**l**): Gastrocnemius lateralis (Gas_lat) muscle activation during stance phase of running. Note. EMG and activations were normalized from zero to one for each subject based upon the minimum and maximum values over the stance phase.

The model simulated muscle activation was also reported from zero to one, with zero representing no muscle activation and one representing full muscle activation. The comparison results are shown in Figure 3 and were in agreement between the predicted muscle activation and sEMG during the stance phase, the predicted results were also compared with previous findings and showed good consistency [29]. Joint angles and moments were compared with a public dataset [30], muscle forces and joint reaction forces were compared against literature values [19].

### 2.6. Data and Statistical Analysis

The joint angles, moments and predicted muscle forces and joint contact forces during the stance phase for each walking, jogging and running trail from heel strike to toe-off on both legs were normalized to 101 data points from 0% to 100%. The joint angles (degrees) and moments (Nm/kg) were reported including the sagittal plane, hip, knee, ankle and the frontal plane subtalar. The lower limb muscle forces and joint contact forces presented for the hip, knee and ankle joints were normalized to body weight (BW). The stance phase of walking was sub-divided into a loading response (0~20%), mid-stance (20~50%), push-off (50~100%) [31]. The stance phase of jogging and running were sub-divided into initial contact (0~50%), mid-stance (~50%~) and push-off (50~100%) as indicated in previous studies [32]. Data normality was checked using the Shapiro-Wilks test prior to statistical analysis. To detect significant differences between the normalized waveforms of involved and uninvolved sides, the open-source (www.spm1d.org) one-dimensional statistical parametric mapping was (SPM1d 0.4, © Copyright 2019, Todd Pataky, created using Sphinx 2.2.0., Kyoto, Japan) applied to independent two-sample t-tests. The measuring error of this method has been previously reported to be less than 0.01, and the retest reliability has been established with an intraclass correlation efficient (ICC) of 0.97. Random field theory (RFT) outlined by Pataky et al., (2013) was employed to determine the potential statistical significance differences between the two sides for biomechanical variables over the whole stance phase in MatLab (R2018a, Mathworks Inc., Natick, MA, USA) [33]. Significance was set at *p* < 0.05.

## 3. Results

### 3.1. Achilles Tendon Length

As shown in Table 1, the gastrocnemii and soleus ATL were found to be significantly longer on the involved side compared to the uninvolved side. The gastrocnemii ATL was 233.0 ± 6.3mm on the involved side compared with 210.4 ± 7.1mm on the uninvolved side (*p* < 0.01). The soleus ATL was 59.5 ± 4.7mm on the involved side compared with 56.7 ± 4.1mm on the uninvolved side (*p* = 0.13).

### 3.2. Joint Kinematics

As shown in Table 2, the peak values of joint kinematics compared between the involved side and the uninvolved side were found significant differences in most of the parameters, especially in the knee flexion, ankle dorsiflexion/plantarflexion, and subtalar eversion. The knee flexion and ankle plantarflexion degrees were decreased in the involved side compared to the uninvolved side.

**Figure 4:**
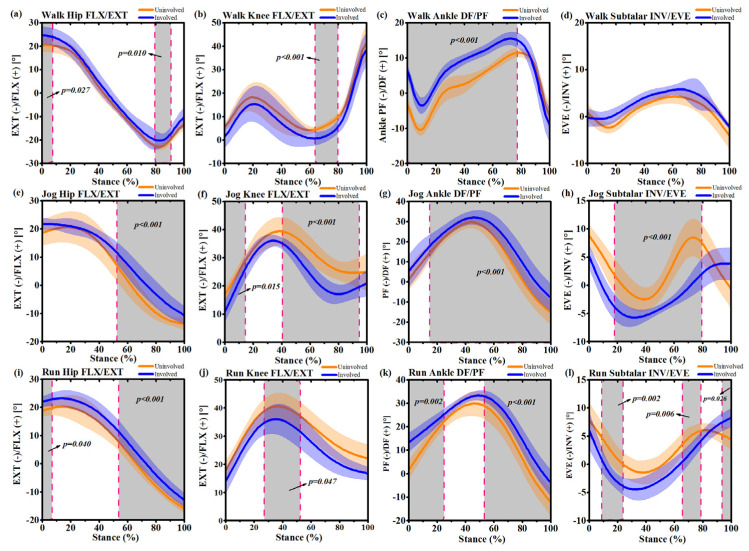
Mean and standard deviation lower extremity joint angle waveforms over stance phase (100%) for the uninvolved side (yellow) and involved side (blue) during self-selected speed walking, jogging and running. (**a**): Hip joint flexion/extension (FLX/EXT) during stance phase of walking; (**b**): Knee joint flexion/extension (FLX/EXT) during stance phase of walking; (**c**) Ankle joint dorsiflexion/plantarflexion (DF/PF) during stance phase of walking; (**d**) Subtalar joint inversion/eversion (INV/EVE) during stance phase of walking; (**e**): Hip joint flexion/extension (FLX/EXT) during stance phase of jogging; (**f**): Knee joint flexion/extension (FLX/EXT) during stance phase of jogging; (**g**) Ankle joint dorsiflexion/plantarflexion (DF/PF) during stance phase of jogging; (**h**) Subtalar joint inversion/eversion (INV/EVE) during stance phase of jogging; (**i**): Hip joint flexion/extension (FLX/EXT) during stance phase of running; (**j**): Knee joint flexion/extension (FLX/EXT) during stance phase of running; (**k**) Ankle joint dorsiflexion/plantarflexion (DF/PF) during stance phase of running (**l**) Subtalar joint inversion/eversion (INV/EVE) during stance phase of running. Grey shaded regions on graphs indicate a significant difference between the two sides (*p* < 0.05) from SPM1d (one-dimensional Statistical Parametric Mapping) analyses.

During self-selected speed walking, the hip flexion angle of the involved side increased at 0%~7% (*p* = 0.027) and 79%~90% (*p* = 0.010) (Figure 4a). The knee flexion angle of the uninvolved side increased at 63%~80% (*p* < 0.001) (Figure 4b). The ankle dorsiflexion angle of the involved side increased at 0~78% (*p* < 0.001) (Figure 4c). During self-selected speed jogging, the hip flexion angle of the involved side increased at 52%~100% (*p* < 0.001) (Figure 4e). The knee flexion angle of the uninvolved side increased at 0~14% (*p* = 0.015) and 41%~95% (*p* < 0.001) (Figure 4f). The ankle dorsiflexion angle of the involved side increased at 13%~100% (*p* < 0.001) (Figure 4g). The subtalar inversion angle of the involved side decreased at 19~80% (*p* < 0.001) (Figure 4h). During self-selected speed running, the hip flexion angle of the involved side increased at 0~7% (*p* = 0.040) and 53%~100% (*p* < 0.001) (Figure 4i). The knee flexion angle of the uninvolved side increased at 28%~52% (*p* = 0.047) (Figure 4j). The ankle dorsiflexion angle of the involved side increased at 0~26% (*p* = 0.002) and 53%~100% (*p* < 0.001) (Figure 4k). The decreased subtalar joint inversion angle of the involved side was observed to be between 8%~23% (*p* = 0.002) and 65%~78% (*p* = 0.006), while the increased subtalar inversion angle was observed to be 93%~100% (*p* = 0.026) of the involved side compared with the uninvolved side (Figure 4l).

### 3.3. Joint Moments

As shown in Table 3, the main differences of the joint moments were found in ankle plantarflexion and subtalar eversion between the involved and the uninvolved sides. The ankle plantarflexion moments in the involved side were significantly decreased, while the subtalar eversion moments were significantly increased during jogging and running.

**Figure 5:**
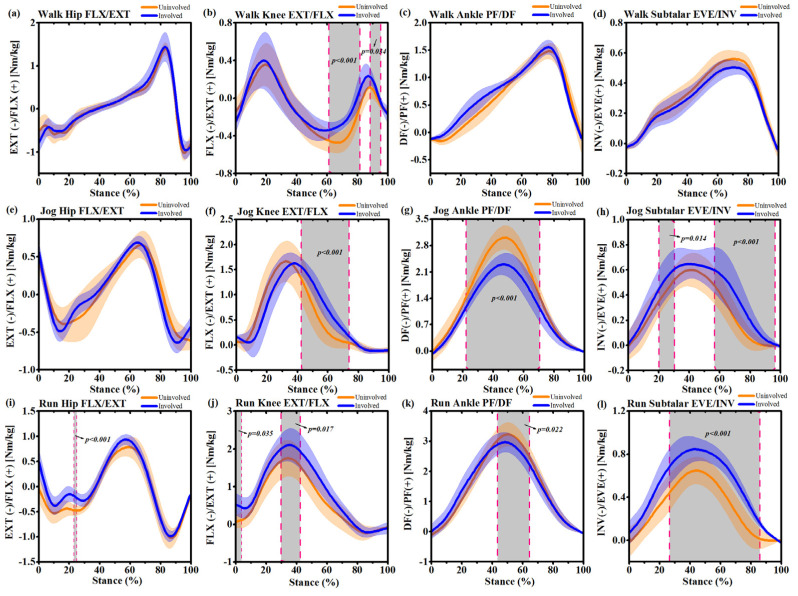
Mean and standard deviation of lower extremity joint moment waveforms over stance for the uninvolved side (yellow) and involved side (blue) during self-selected speed walking, jogging and running. (**a**): Hip joint flexion/extension (FLX/EXT) moment during stance phase of walking; (**b**): Knee joint flexion/extension (FLX/EXT) moment during stance phase of walking; (**c**) Ankle joint dorsiflexion/plantarflexion (DF/PF) moment during stance phase of walking; (**d**) Subtalar joint inversion/eversion (INV/EVE) moment during stance phase of walking; (**e**): Hip joint flexion/extension (FLX/EXT) moment during stance phase of jogging; (**f**): Knee joint flexion/extension (FLX/EXT) moment during stance phase of jogging; (**g**) Ankle joint dorsiflexion/plantarflexion (DF/PF) moment during stance phase of jogging; (**h**) Subtalar joint inversion/eversion (INV/EVE) moment during stance phase of jogging; (**i**): Hip joint flexion/extension (FLX/EXT) moment during stance phase of running; (**j**): Knee joint flexion/extension (FLX/EXT) moment during stance phase of running; (**k**) Ankle joint dorsiflexion/plantarflexion (DF/PF) moment during stance phase of running (**l**) Subtalar joint inversion/eversion (INV/EVE) moment during stance phase of running. Grey shaded regions on graphs indicate a significant difference between the two sides (*p* < 0.05) from SPM1d analyses.

During the walking movement, no significant differences were found between the involved and uninvolved sides for the hip joint (Figure 5a). Reduced knee flexion moment was observed in the involved side between 62%~81% (*p* < 0.001), whereas the knee extension moment of the involved side increased at 90%~95% (*p* = 0.034) (Figure 5b). During jogging movements, the knee extension moment of the involved side increased at 49%~74% (*p* < 0.001) (Figure 5f). The ankle plantarflexion moment of the uninvolved side increased at 22%~70% (*p* < 0.001) (Figure 5g). The uninvolved side had a reduced subtalar eversion moment during the whole waveform, especially during 20%~29% (*p* = 0.014) and 56%~96% (*p* < 0.001) of the stance phase (Figure 5h). During running movements, the hip extension moment of the involved side decreased between 23%~25% (*p* < 0.001) (Figure 5i). The knee extension moment of the involved side increased at 0~3% (*p* = 0.035) and 30%~42% (*p* = 0.017) (Figure 5j). The ankle plantarflexion moment of the uninvolved side increased at 44%~63% (*p* = 0.022) (Figure 5k). The subtalar eversion moment of the uninvolved side decreased at 27%~85% (*p* < 0.001) (Figure 5l).

### 3.4. Muscle Forces

As shown in Table 4, for the quadriceps femoris, the vastus medialis, vastus lateralis and vastus intermedius muscle forces were found increased in the uninvolved side during walking movement, while decreased during jogging and running movements. As shown in Table 5, for the triceps surae, the gastrocnemius medialis muscle forces were decreased in the involved side during walking, jogging and running. The gastrocnemius lateralis muscle forces were decreased during both walking and jogging in the involved side, while the soleus muscle force was only found a decrease in the involved side during walking movement.

**Figure 6:**
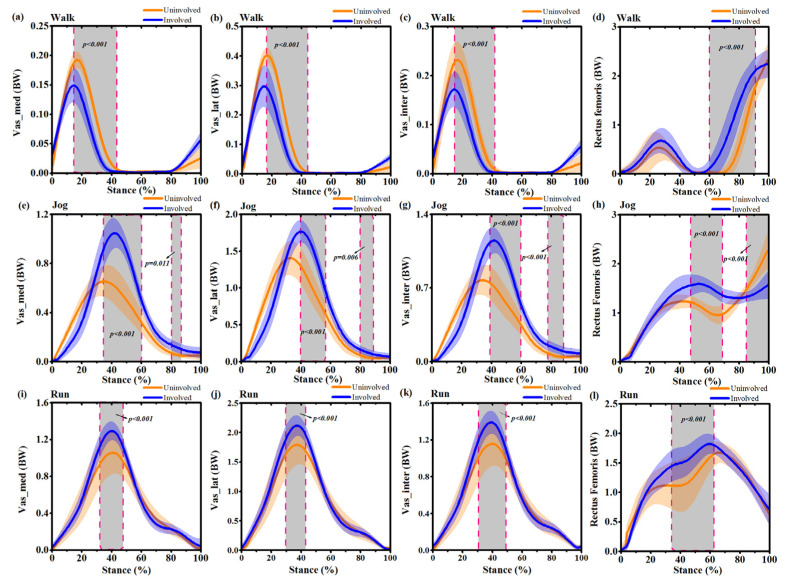
Mean and standard deviation of estimated vas_med (vastus medialis), vas_lat (vastus lateralis), vas_inter (vastus intermedius) and rectus femoris muscle-tendon forces waveforms over stance between the uninvolved side (yellow) and involved side (blue) during walking, jogging and running. (**a**): vas_med muscle-tendon forces during stance phase of walking; (**b**): vas_lat muscle-tendon forces during stance phase of walking; (**c**) vas_inter muscle-tendon force during stance phase of walking; (**d**) rectus femoris muscle-tendon force during stance phase of walking; (**e**): vas_med muscle-tendon forces during stance phase of jogging; (**f**): vas_lat muscle-tendon forces during stance phase of jogging; (**g**) vas_inter muscle-tendon force during stance phase of jogging; (**h**) rectus femoris muscle-tendon force during stance phase of jogging; (**i**): vas_med muscle-tendon forces during stance phase of running; (**j**): vas_lat muscle-tendon forces during stance phase of running; (**k**) vas_inter muscle-tendon force during stance phase of running; (**l**) rectus femoris muscle-tendon force during stance phase of running. Grey shaded regions on graphs indicate a significant difference between the two sides (*p* < 0.05) from SPM1d analyses.

**Figure 7:**
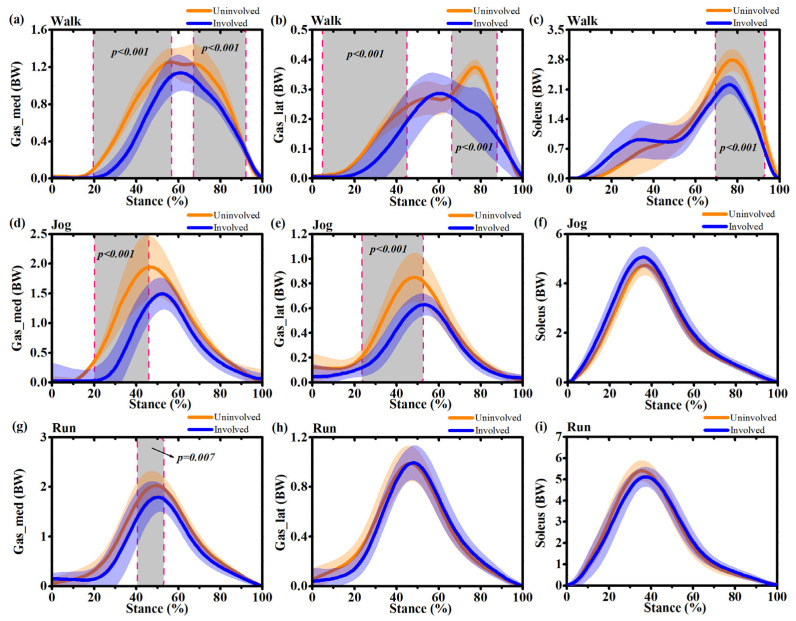
Mean and standard deviation of estimated gas_med (gastrocnemius medialis), gas_lat (gastrocnemius lateralis) and soleus muscle-tendon forces waveforms over stance between the uninvolved side (yellow) and involved side (blue) during walking, jogging and running. (**a**): gas_med muscle-tendon forces during stance phase of walking; (**b**) gas_lat muscle-tendon forces during stance phase of walking; (**c**) soleus muscle-tendon forces during stance phase of walking; (**d**): gas_med muscle-tendon forces during stance phase of jogging; (**e**) gas_lat muscle-tendon forces during stance phase of jogging; (**f**) soleus muscle-tendon forces during stance phase of jogging; (**g**): gas_med muscle-tendon forces during stance phase of running; (**h**) gas_lat muscle-tendon forces during stance phase of running; (**i**) soleus muscle-tendon forces during stance phase of running. Grey shaded regions on graphs indicate a significant difference between the two sides (*p* < 0.05) from SPM1d analyses.

The SPM1d analysis of the muscle force data found significant differences in all three movements (Figure 6 and Figure 7). The vastus medialis (vas_med), vastus lateralis (vas_lat) and vastus intermedius (vas_inter) force waveforms were found to have similar trajectory changes during walking, jogging and running, respectively. During walking, the uninvolved side was found to increase in vas_med (19%~46%, *p* < 0.001), vas_lat (17%~43%, *p* < 0.001) and vas_inter (16%~42%, *p* < 0.001) forces compared to the involved side (Figure 6a–c). While the involved side recorded increased vas_med (26%~47%, *p* < 0.001), vas_lat (30%~42%, *p* < 0.001) and vas_inter (30%~44%, *p* <0.001) forces during running (Figure 6i–k). During the mid-stance and push-off phases of jogging movements, the vas_med, vas_lat and vas_inter forces showed increases in the involved side (Figure 6e–g). The rectus femoris force during walking was observed higher in the involved side at the push-off phase (60%~85%, *p* < 0.001). During jogging, the rectus femoris force of the involved side increased at 44%~64% (*p* < 0.001), whereas it decreased at 84%~100% (*p* < 0.001). The rectus femoris force was significantly (*p* < 0.001) greater from 34%~62% of the stance phase in the involved side during running (Figure 6l). The three Achilles tendon related triceps surae muscles were also significantly different between side-to-side during the three movements. During walking, the gastrocnemius medialis (gas_med) force of the uninvolved side increased at 20%~48% (*p* < 0.001) and 68%~92% (*p* < 0.001) (Figure 7a). The gastrocnemius lateralis (gas_lat) force of the uninvolved side increased at 5%~44% (*p* < 0.001) and 68%~88% (*p* < 0.001) (Figure 7b). The soleus force was found to decrease in the involved side during the push-off phase (70%~93%, *p* < 0.001) during walking (Figure 7c). During jogging movements, gas_med and gas_lat force waveforms were found completely similar during the whole stance phase. The gas_med and gas_lat forces of the uninvolved side were significantly higher than the involved side at 20%~43% (*p* < 0.001) and 22%~51% (*p* < 0.001), respectively. During running, the gas_med force was significantly greater from 40%~52% (*p* = 0.007) in the involved side (Figure 7g), while no significant differences were found in gas_lat force between the involved and uninvolved sides. The soleus forces during both jogging and running were also non-significant between the involved and uninvolved sides.

### 3.5. Joint Reaction Forces

As shown in Table 6, the hip joint reaction forces were increased on the involved side during walking and jogging. The knee joint reaction forces were increased in the involved side during walking and jogging, while decreased during running. The ankle joint reaction forces were decreased in the involved side during walking and running, while increased during jogging.

**Figure 8:**
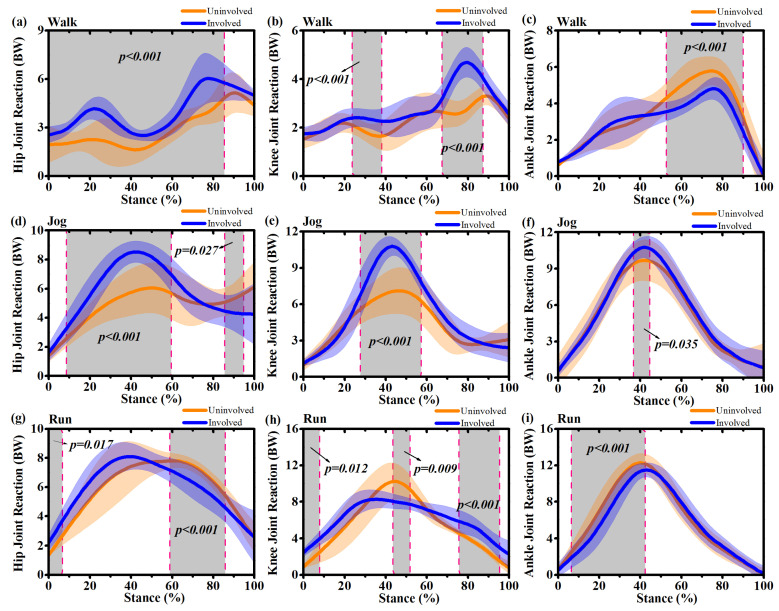
Mean and standard deviation of estimated joint reaction forces waveforms over stance at the hip, knee and ankle joints for uninvolved side (yellow) and involved side (blue) during walking, jogging and running. (**a**): Hip joint reaction forces during stance phase of walking; (**b**) Knee joint reaction forces during stance phase of walking; (**c**) Ankle joint reaction forces during stance phase of walking; (**d**): Hip joint reaction forces during stance phase of jogging; (**e**) Knee joint reaction forces during stance phase of jogging; (**f**) Ankle joint reaction forces during stance phase of jogging; (**g**): Hip joint reaction forces during stance phase of running; (**h**) Knee joint reaction forces during stance phase of running; (**i**) Ankle joint reaction forces during stance phase of running. Grey shaded regions on graphs indicate a significant difference between the two sides (*p* < 0.05) from SPM1d analyses.

During walking, the hip joint reaction force of the involved side increased at 0%~85% (*p* < 0.001) (Figure 8a). The knee joint reaction force of the involved side increased from 24% to 39% (*p* < 0.001) and from 69% to 88% (*p* < 0.001) (Figure 8b). The uninvolved side showed increased ankle joint force from 48%~90% (*p* < 0.001) compared to the involved side (Figure 8c). During jogging, increased hip joint reaction forces of the involved side were observed during the mid-stance at 10%~60% (*p* < 0.001), while decreased hip joint reaction forces of the involved side were found during push-off between 86%~95% (*p* = 0.027) (Figure 8d). Increased knee and ankle joint reaction forces of the involved side were also found during the mid-stance at 29%~59% (*p* < 0.001) and 38%~44% (*p* = 0.035), respectively (Figure 8e, 8f). During running movements, increased hip joint contact forces of the involved side were observed at 0%~7% (*p* = 0.017) during initial contact, while decreases were observed at 59%~85% (*p* < 0.001) during the push-off phase. On the knee joint, there were significant differences at three separate instances in the stance phase, increased knee contact forces of the involved side during initial contact (0%~8%, *p* = 0.012) and push-off (77%~97%, *p* < 0.001) and decreased knee contact force of the involved side during mid-stance (43%~52%, *p* = 0.009) (Figure 8h). The ankle joint force of the uninvolved side increased at 7%~43% during initial contact (*p* < 0.001) (Figure 8i).

## 4. Discussion

This study integrates an in vivo gait analysis, ultrasound imaging based subject-specific AT repaired lower limb musculoskeletal model to investigate if geometry modification of the AT was present after 2 years operative treatment of the unilateral ATR. The study also investigated if as a consequence, lower limb kinematics, kinetics, and muscular contributions were different between the involved and uninvolved legs. In the current study, the mean ATRS value (83.7) of these patients reported fairly normal physical activity and function and recovered well after two years ATR [21]. As hypothesized, a discrepancy was found between the patient-reported outcomes and the tests, which showed large impairment and significant functional deficits with an elongated AT persisting on the injured side in patients with a surgically repaired Achilles tendon at two years. The previous findings showed higher AT length 2–6 years after surgical repair on the previously injured leg, which supports our first hypothesis [34].

Gait analysis has been used to identify intrinsic gait-related risk factors, which revealed altered ankle joint sagittal plane kinematics and plantarflexion moments compared to the uninjured side [35]. Increased ankle dorsiflexion was accompanied by decreased plantarflexion and was observed almost during the whole stance phase of walking, jogging and running movements. This has also been found in previous studies [4,36]. It has been suggested that increased ankle dorsiflexion may reveal a sustained eccentric dynamic control deficit even 4.5 years after an ATR [37]. An association between increased ankle dorsiflexion and consistent ankle plantar flexor eccentric contraction deficits two years after an ATR was also reported [38]. The plantarflexion moment of the involved limb was lower during the mid-stance phase of jogging and running. The intra-patient AT length and stiffness asymmetries were found related to deficits in plantarflexion moments during gait in a previous study [34]. While this study failed to find any difference between the involved and uninvolved sides during normal walking. This discrepancy in such results could be attributed to the physical activity level and follow-up time in this study.

Willy et al., suggested that the side to side deficits and discrepancies were larger in ATR patients with high-demand and higher angular velocity activities, i.e., jogging and hopping compared with walking [39]. The decreased plantarflexion angle during the push-off phase in jogging and running movements of the injured leg suggested decreased triceps surae contribution in the center of mass displacement during locomotion [40]. The ankle joint kinematic and kinetic data obtained in this study suggest that the ankle plantar flexor function is more impaired during higher-demand movements and higher end-range ankle joint plantarflexion after an ATR. A previous study showed the plantar flexor muscles suffered from a functional deficit in patients with an ATR even more than two years after surgery [9]. It was speculated that the increased ankle dorsiflexion degree accompanied by a decreased plantar flexion moment may be a compensatory mechanism of insufficient ankle dorsal flexor function and the anomalous co-contraction of tibialis anterior and calf muscles, which has been confirmed in previous gait analysis with 41 ATR patients one year after surgery [41].

As hypothesized, the joint reaction forces will decrease, while the results of this study do not support this hypothesis. While this study found that increased knee joint loading associated with decreased ankle joint function was present in patients two years after an ATR. It was reported that if the AT length of the involved side increased, the muscle fascicle length will decrease by an equal amount [8]. The AT is not a contractile tissue compared to muscle and can’t provide positive work to produce human motion. Thus the shorter triceps surae fascicles in the involved limb may promote a deficit in plantar flexor power generation [42]. The compromised plantar flexor function of the injured leg following an ATR may compensate by increased work done by the ipsilateral knee joint [43]. In this study, increased knee extension angles and elevated knee joint moments were found in the involved limb during all activities. Furthermore, increased knee joint contact forces were also present during walking, jogging and running except in the mid-stance phase of running movements. This study showed increased knee extension in the involved limb during initial contact and push-off phases of the jogging and mid-stance phase of running. The increased knee extension during jogging and running maybe a potential regulatory mechanism to ensure sufficient elongated triceps surae complex tension [44]. A previous study supports the findings of this study, which found a compensation mechanism of the overextension knee joint during running against the AT elongation and deficit of gastro-soleus muscle-tendon complex [10]. The calf muscle crosses two joints, which play an important role to prevent knee overextension and anterior knee laxity as a stabilizer. Thus the deficit and weakness of the calf muscle in the involved limb may place the knee in an overextension position and at risk for further injury [45]. It was found that the hip joint was more flexed during the push-off phase of jogging and running, which may be a compensation mechanism following overextension of the knee joint to prevent injury risk during high-demand tasks.

The resultant pattern of greater knee extension moment and knee joint contact forces in the involved limb may result in an overloaded knee extensor mechanism. The increased knee joint loading may be attributed to weakened ankle plantar flexor function and elongation of the AT during higher demand tasks like jogging, running and even basic locomotion like walking. The findings of this study were inconsistent with earlier works that showed significantly greater patellofemoral joint loading in the injured leg only during high-demand activities but not during walking in patients after surgical ATR repair [41,43]. The reason for the inconsistency may be due to the different physical activity levels and follow-up time of the subjects. The average ATRS value of the previous study is 87.0 compared with 83.7 in this study, and the follow-up time of the previous study was 6.2 years on average compared to two years in our study. Probably, the AT of the patients in this study were still in the remodeling stage and the symptoms and functions will reach a plateau two years after ATR [46]. The greater knee/patellofemoral joint loading was reported positively correlated with patellofemoral pain and even knee osteoarthritis in previous studies [47,48]. Further study should assess the knee symptoms in patients recovering from an ATR.

In the frontal plane, the subtalar joint angle and moment patterns were also changed between the involved and uninvolved sides. During jogging and running, the ankle joint was found more everted combined with greater subtalar eversion moment during almost the whole stance phase. The ankle joint over-eversion was observed associated with several running-related injuries, including medial tibial stress syndrome and more knee injuries [49]. The deficit of plantar flexor muscle operation in the sagittal plane may be substituted by increased loading in the frontal plane of the lower limb joints [50]. Consistently, Jandacka et al. reported that a weakened and elongated AT may be the main reason causing altered sagittal plane knee and ankle joint kinematics and increasing lower extremity frontal-plane loading as a consequence [43].

To the extent of our knowledge, this is the first study to investigate the side-to-side muscular contribution during normal activities via subject-specific OpenSim musculoskeletal modeling after a unilateral ATR. The knee extension femur muscle strength was found conversely increased during jogging and running. While during walking, only the rectus femoris force was found higher in the involved side during the push-off phase, the vas_med, vas_lat and vas_inter forces were still showed deficits in the involved side. In accordance with the present results, which have demonstrated that significantly greater knee joint power was observed in the involved side as compensation for reduced plantar flexor function during high-demand activities only but was not observed during walking [51]. The greater knee extension muscular forces in the involved side in this study were also following a higher knee extension degree and moments during jogging and running.

In the present study, it was found that significant lower limb muscle strength deficits during normal movements persist on the injured side two years after an acute ATR. The gastrocnemius and soleus muscle forces were found significantly decreased in the involved side during the stance phase of walking, jogging and running. Thus, the results do not support the hypothesis of the triceps surae muscle forces will increase to compensate for the additional slack length and decreased force transmission capacity of the AT.

There are several possible explanations for this result. The first interpretation was that with the elongated AT in the involved side, the shank muscles are atrophied, the AT properties may tend to more compliant and hysteresis, which may hurt the stretch-shortening cycle and muscle-tendon interaction during the stance phase of the involved limb [52,53,54]. The second interpretation was when the ankle joint in plantar flexion position during the push-off phase, the triceps surae muscle was in a shortened posture and below the optimal angle for force generatio. These findings are in accordance with a study that reported the additional slack of the AT may lead to decreased plantarflexion strength [5]. The weakened plantar flexor muscles on the involved side and asymmetry may overload the muscle-tendon complex in the uninvolved side. Furthermore, the plantar flexor muscle deficit may affect the mediolateral acceleration of the center of mass based on a previous study and thus disturb the frontal plane ankle stability of the injured side. This may be an additional explanation for the increased ankle joint eversion in the involved side in this study. The increased AT length and decreased triceps surae strength to stretch the AT indicate that the functional deficit in the involved side may be primarily caused by anatomical alterations of the tendon. Thus, the calf muscle asthenia may be due to inefficacies in force transmission capacity across the joint.

Several limitations should not be ignored in the current study. The first limitation relates to the small sample size in this study. However, we have still observed significant musculoskeletal abnormalities between the involved and uninvolved sides in patients recovering from an ATR. The second limitation was the lack of a healthy control group and to assess inter-limb differences, while an inter-limb gait function test may be a conservative assessment after an ATR. The third limitation of this study was that the movement speed (about 3.5 m/s during running) during testing in the laboratory was relatively slow compared to the real outdoor activities. The ATR mostly occurred during a rapid plantar flexor eccentric loading phase. Thus, further studies should investigate lower limb musculoskeletal functional differences during high-intensity movements like cutting and landing in both ATR patients and uninjured subjects.

## 5. Conclusions

This study provides subject-specific lower limb musculoskeletal models on six patients recovering from a unilateral ATR based on ultrasound imaging data, for the prediction of joint kinematics, joint moments, muscle strength and joint reaction forces during daily activities. It seems the increased AT length is associated with persistent triceps surae muscle strength deficits and decreased plantarflexion moments after surgical repair of ATR during walking, jogging and running. The greater knee extension femur muscle forces combined with increased knee extension moments and higher joint reaction forces during normal daily activities were predicted as a compensation strategy for ankle impairment and plantar flexor function deficit. In the frontal plane, the elevated subtalar joint eversion degrees and moments in the involved side may be due to weakened plantar flexor muscle and perhaps disturb the frontal plane ankle stability, which is confirmed by greater knee loading in the medial part. Speculation of this study is that patients after an ATR may suffer from increased knee overuse injury risk compared to healthy controls. Future studies should confirm these results with bigger sample size and develop subject-specific musculoskeletal knee joint models to predict medial and lateral knee/tibiofemoral contact forces during explosive movements like cutting, landing and hopping of the affected limb with participants after an ATR.

## Figures and Tables

**Table 1:** Subject AT length details. The gastrocnemii and soleus AT lengths (ATL) of each subject were measured for both the involved and uninvolved sides.

Subject	Gastrocnemii ATL (mm)				Soleus ATL (mm)			
	IS	US	ISATL/USATL	USATL/ISATL	IS	US	ISATL/USATL	USATL/ISATL
01	228.6	201.4	1.14	0.88	56.1	54.2	1.04	0.97
02	242.2	221.3	1.09	0.91	62.2	58.6	1.06	0.94
03	235.5	212.2	1.11	0.90	53.1	51.2	1.04	0.96
04	236.1	205.7	1.15	0.87	66.5	63.3	1.05	0.95
05	224.5	207.2	1.08	0.92	58.7	55.6	1.06	0.95
06	231.2	214.8	1.08	0.93	60.4	57.5	1.05	0.95

Note. “ATL” equals to “Achilles tendon length”, “IS” equals to “involved side”, “US” equals to “uninvolved side”, “ISATL” equals to “involved side Achilles tendon length”, “USATL” equals to “uninvolved side Achilles tendon length”.

**Table 2:** The peak joint kinematics of the involved side (IS) and the uninvolved side (US) during walking, jogging and running movements.

	Joint kinematics (°)	IS	US	*p*-Value
Walking	Hip flexion	25.3 ± 3.4	20.1 ± 2.9	<0.001
	Hip extension	18.3 ± 2.6	22.1 ± 1.8	0.03
	Knee flexion	37.4 ± 4.2	39.1 ± 3.7	0.42
	Ankle dorsiflexion	16.2 ± 2.3	11.3 ± 2.6	<0.001
	Ankle plantarflexion	3.8 ± 2.5	10.1 ± 3.2	<0.001
	Subtalar inversion	5.1 ± 3.3	4.2 ± 2.9	0.37
	Subtalar eversion	1.6 ± 1.1	2.2 ± 2.4	0.09
Jogging	Hip flexion	21.2 ± 3.5	19.3 ± 4.4	0.28
	Hip extension	10.1 ± 3.1	13.2 ± 3.9	0.02
	Knee flexion	35.2 ± 2.9	39.7 ± 3.6	0.01
	Ankle dorsiflexion	32.6 ± 4.1	27.3±2.9	<0.001
	Ankle plantarflexion	5.3 ± 2.8	12.4±3.9	<0.001
	Subtalar inversion	4.2 ± 3.2	8.1 ± 4.5	<0.001
	Subtalar eversion	5.4 ± 3.4	2.1 ± 3.3	<0.001
Running	Hip flexion	22.3 ± 3.3	18.9±3.1	0.03
	Hip extension	11.2 ± 2.9	13.6±2.8	0.01
	Knee flexion	35.1 ± 5.2	40.8±4.9	0.04
	Ankle dorsiflexion	32.3 ± 4.1	29.5±4.5	0.02
	Ankle plantarflexion	3.2 ± 2.9	10.1±4.3	<0.001
	Subtalar inversion	6.2 ± 2.8	5.4±3.3	0.47
	Subtalar eversion	4.6 ± 2.5	1.3±2.2	0.01

Note. “IS” equals to “involved side”, “US” equals to “uninvolved side”.

**Table 3:** The peak joint moments of the involved side (IS) and the uninvolved side (US) during walking, jogging and running movements.

	Joint Moments (Nm/kg)	IS	US	*p*-Value
Walking	Hip flexion	1.42 ± 0.15	1.39 ± 0.12	0.87
	Knee flexion	0.26 ± 0.14	0.42 ± 0.21	<0.001
	Knee extension	0.22 ± 0.18	0.13 ± 0.20	<0.001
	Ankle plantarflexion	1.51 ± 0.16	1.50 ± 0.18	0.92
	Subtalar eversion	0.47 ± 0.18	0.52 ± 0.22	0.21
Jogging	Hip flexion	0.71 ± 0.32	0.69 ± 0.26	0.45
	Knee extension	1.58 ± 0.38	1.60 ± 0.42	0.33
	Ankle plantarflexion	2.21 ± 0.42	2.95 ± 0.56	<0.001
	Subtalar eversion	0.64 ± 0.33	0.58 ± 0.25	0.02
Running	Hip flexion	0.93 ± 0.22	0.88 ± 0.19	0.41
	Knee extension	2.12 ± 0.44	1.74 ± 0.39	<0.001
	Ankle plantarflexion	2.92 ± 0.33	3.23 ± 0.31	0.02
	Subtalar eversion	0.85 ± 0.25	0.62 ± 0.27	<0.001

Note. “IS” equals to “involved side”, “US” equals to “uninvolved side”.

**Table 4:** The peak quadriceps femoris muscle forces of the involved side (IS) and the uninvolved side (US) during walking, jogging and running movements, BW equals body weight.

	Muscle forces (BW)	IS	US	*p*-Value
Walking	Vastus medialis	0.15 ± 0.04	0.19 ± 0.02	<0.001
	Vastus lateralis	0.29 ± 0.05	0.40 ± 0.02	<0.001
	Vastus intermedius	0.17 ± 0.04	0.23 ± 0.03	0.01
	Rectus femoris	2.24 ± 0.33	2.27 ± 0.28	0.43
Jogging	Vastus medialis	0.85 ± 0.12	0.68 ± 0.17	<0.001
	Vastus lateralis	1.76 ± 0.38	1.44 ± 0.45	0.01
	Vastus intermedius	1.15 ± 0.22	0.82 ± 0.16	<0.001
	Rectus femoris	1.57 ± 0.19	2.23 ± 0.28	<0.001
Running	Vastus medialis	1.32 ± 0.11	1.05 ± 0.24	<0.001
	Vastus lateralis	2.08 ± 0.23	1.77 ± 0.41	<0.001
	Vastus intermedius	1.38 ± 0.12	1.15 ± 0.33	<0.001
	Rectus femoris	1.69 ± 0.24	1.58 ± 0.27	0.02

Note. “IS” equals to “involved side”, “US” equals to “uninvolved side”.

**Table 5:** The peak triceps surae muscle forces of the involved side (IS) and the uninvolved side (US) during walking, jogging and running movements, BW equals body weight.

	Muscle forces (BW)	IS	US	*p*-Value
Walking	Gastrocnemius medialis	1.12 ± 0.43	1.26 ± 0.36	0.02
	Gastrocnemius lateralis	0.28 ± 0.08	0.37 ± 0.03	<0.001
	Soleus	2.12 ± 0.26	2.78 ± 0.31	<0.001
Jogging	Gastrocnemius medialis	1.51 ± 0.32	1.92 ± 0.46	0.01
	Gastrocnemius lateralis	0.63 ± 0.23	0.84 ± 0.46	<0.001
	Soleus	4.95 ± 0.44	4.88 ± 0.39	0.52
Running	Gastrocnemius medialis	1.81 ± 0.32	2.02 ± 0.29	0.03
	Gastrocnemius lateralis	0.86 ± 0.21	0.87 ± 0.18	0.94
	Soleus	5.15 ± 0.92	5.19 ± 0.76	0.23

Note. “IS” equals to “involved side”, “US” equals to “uninvolved side”.

**Table 6:** The peak joint reaction forces of the involved side (IS) and the uninvolved side (US) during walking, jogging and running movements.

	Joint reaction forces (BW)	IS	US	*p*-Value
Walking	Hip	5.92 ± 1.03	4.86 ± 1.11	0.01
	Knee	4.67 ± 0.86	3.12 ±0.97	<0.001
	Ankle	4.39 ± 0.94	5.81 ±1.21	<0.001
Jogging	Hip	8.22 ± 0.68	5.94 ±1.32	<0.001
	Knee	11.22 ± 2.33	6.85 ±1.78	<0.001
	Ankle	10.62 ± 2.46	9.21 ± 3.85	0.04
Running	Hip	7.94 ± 1.31	7.88 ± 2.44	0.36
	Knee	8.05 ± 1.05	10.24 ± 1.97	0.01
	Ankle	10.54 ± 1.54	11.96 ± 1.32	0.03

Note. “BW” equals to “body weight”.
